# Comparison of fecal pyruvate kinase isoform M2 and calprotectin in acute diarrhea in hospitalized children

**DOI:** 10.1038/srep04769

**Published:** 2014-04-24

**Authors:** Elzbieta Czub, Jan K. Nowak, Jerzy Moczko, Aleksandra Lisowska, Aleksandra Banaszkiewicz, Tomasz Banasiewicz, Jaroslaw Walkowiak

**Affiliations:** 1 Child and Mother Specialist Hospital, Poznan, Poland; 2 Department of Pediatric Gastroenterology and Metabolic Diseases, Poznan University of Medical Sciences, Poznan, Poland; 3 Department of Informatics and Statistics, Poznan University of Medical Sciences, Poznan, Poland; 4 Department of Pediatric Gastroenterology and Nutrition, Medical University of Warsaw, Warsaw, Poland; 5 Department of General, Gastroenterological and Endocrinological Surgery, Poznan University of Medical Sciences, Poznan, Poland

## Abstract

Fecal concentrations of pyruvate kinase isoform M2 (M2-PK) and calprotectin (FC) serve as biomarkers of inflammation of gastrointestinal mucosa. The value of M2-PK in discriminating between patients with viral and bacterial acute diarrhea (AD) is currently unknown. We analyzed M2-PK and FC concentrations in fifty hospitalized children with AD (29 of which were caused by rotavirus and 21 by *Salmonella enteritidis*) as well as 32 healthy subjects. There was no difference in the areas under the receiver operating characteristic curves plotted for the two tests in differentiating rotaviral from bacterial AD. The sensitivity and specificity of M2-PK at optimal cut-off (20 U/g) were 75.9% and 71.4%, respectively. M2-PK and FC had similar values in distinguishing between children with AD caused by rotavirus and *Salmonella enteritidis*. The performance of both tests in hospitalized patients did not meet the needs of everyday clinical practice. Moreover, no advantage of fecal tests over the measurement of CRP was documented.

Pyruvate kinase embryonic isoform M2 (M2-PK) is an enzyme responsible for phosphate group transfer in glycolysis^1^. It has been shown that M2-PK dimers and tetramers are present in proliferating cells in different tissues^2^. Because M2-PK is also present in leukocytes, it can be found in fecal masses that are formed while in contact with the inflamed mucosa of the gastrointestinal tract^1^. As M2-PK stability is high, its concentrations in stools can reflect gastrointestinal inflammation. Two main proposed uses of M2-PK in gastroenterology are the assessment of inflammatory bowel diseases (IBD) severity and cancer screening^3^. We described the potential of M2-PK as a biomarker of pediatric IBD activity^4^.

Calprotectin is a protein of the S100 family that represents over 40% of the proteins found in the neutrophils' cytosol. Calprotectin can halt bacterial growth, playing an important role in non-specific immune reactions^5^. Concentrations of fecal calprotectin (FC) have been measured in patients with IBD, and it was shown that they may serve a role in disease activity assessment^6^ and relapse prediction^7^. The most recent findings on this topic are summarized in a recent review by Henderson et al.^8^.

In 2008 Shastri et al.^9^ analyzed FC levels in 2383 samples provided by adult patients with acute diarrhea (AD). They observed that FC had a specificity and sensitivity of 83% and 87%, respectively, in identifying patients with positive stool cultures. In 2010, Sýkora et al. investigated FC levels in 66 children with acute gastroenteritis and in 41 healthy controls^10^. Sensitivity and specificity of FC in identifying patients with bacterial cause of gastroenteritis among all enrolled subjects were 93% and 88%, respectively, at a cut-off level of 103.9 μg/g.

So far, the three groups have compared the diagnostic value of M2-PK and FC in IBD^11^,^12^,^13^. However, to date, no studies have been published on the potential of M2-PK and FC in the differential diagnosis of the etiology of AD. The aim of this study was to address this problem by comparing M2-PK and FC in children with AD caused by rotavirus (AD-RV) and *Salmonella enteritidis* (AD-SE).

## Results

M2-PK and FC median concentrations in patients with AD-RV and AD-SE were significantly higher than in HS (p < 0.0001 for all four comparisons; Table 1). The AUC for ROC describing the performance of M2-PK and FC in discriminating between patients with AD-RV and HS were 0.93 (95% CI 0.87–0.99, p < 0.0001) and 0.89 (0.81–0.97, p < 0.0001), respectively.

Identification of patients with AD-SE among HS gave an AUC for ROC of 1.00 (95% CI 0.99–1.00, p < 0.0001) in the case of both M2-PK and FC. The sensitivities and specificities of M2-PK and FC in recognizing children with AD-RV and AD-SE among HS are presented in Table 2.

**Figure 1:**
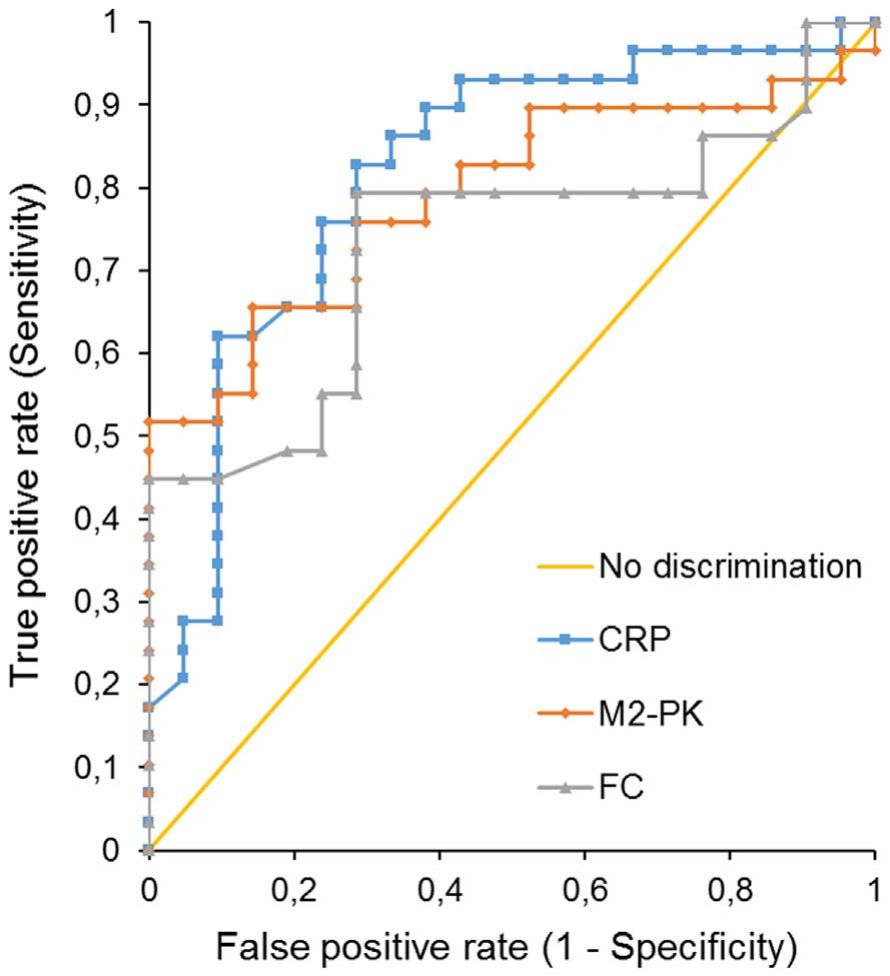
ROC analysis for discrimination between bacterial and viral origin of diarrhea.

The AUC of ROC plotted for M2-PK used in differentiating AD-RV from AD-SE was 0.79 (95% CI 0.67–0.92, p < 0.0001). For the FC, the AUC of the ROC was 0.73 (95% CI 0.59–0.88, p = 0.0006). The difference between these two AUC was not statistically significant (95% CI 0.00–0.12, p = 0.057) (Figure 1). The sensitivity and specificity of M2-PK in discriminating patients with AD-RV from patients with AD-SE were 75.9% and 71.4%, respectively, at a cut-off of 20 U/g. The sensitivity and specificity of FC in this application at a cut-off value of 40.0 μg/mL were 79.3% and 71.4%, respectively. With a specificity set at 1, the sensitivity of M2-PK was 51.7% (cut-off at 10.0 U/g), and the sensitivity of FC equaled 44.8% (cut-off at 15.7 μg/mL).

The AUC of the ROC plotted for CRP used in differentiating AD-RV from AD-SE was 0.82 (95% CI 0.69–0.94, p < 0.0001). No difference between the AUC's for M2-PK and CRP was stated (0,03; 95% CI −0.04–0.09). Interestingly, AUC was significantly larger for CRP than for FC (0.08; 95% CI 0.00–0.18, p = 0.046) (Figure 1).

## Discussion

This is the first study to investigate M2-PK in infectious diarrhea and to compare M2-PK and FC in diarrhea. Each of the two experimental patient groups reflected the main characteristics of AD's two etiologies; that is, AD-RV reflected viral AD and AD-SE did so for bacterial AD.

The main finding is that M2-PK and FC had similar efficacies in discriminating patients with viral and bacterial cause of AD. In comparisons of M2-PK and FC in IBD, both tests also showed similar efficacy^11^,^12^,^13^.

Analysis of the ROC curves showed that the potential value of M2-PK could be greater than FC in differentiating between AD-RV and AD-SE. However, the sensitivities and specificities for both tests were low, e.g. M2-PK at a point of 100% specificity (10 U/g) was 51.7% sensitive in excluding bacterial diarrhea. Thus, both of those markers fail to meet the needs of clinical practice. Moreover, it was documented that the practical value of FC in differentiation between viral and bacterial origin of diarrhea in hospital settings could be lower than that of CRP.

The sensitivity and specificity of FC in identification of patients with AD-RV among all AD patients in this study (79.3% and 71.4%) were lower than observed by Sýkora et al. (92% and 88%)^10^. However, Sýkora et al. analyzed a mixed population of patients from both tertiary and primary care in whom discrepancies between concentrations of FC in bacterial AD and viral AD were greater. Higher concentrations of FC in AD-RV in the present study can be explained by the study's setting parameter, as only hospitalized patients were recruited. Hospitalized AD-RV patients can be assumed to have greater gastrointestinal inflammation than patients with AD-RV treated in primary care settings. The findings of this study are consistent with those obtained by Shastri et al. in a large cohort of adult patients (mean age of 43.8 years). It should be noted that patients recruited by Shastri et al. were also hospitalized in reference centers.

In conclusion, no significant differences were shown in the performance of M2-PK and FC in discriminating between children with AD caused by rotavirus and those with *S. enteritidis*, and further, in identifying them among HS. The two markers did not prove their clinical utility in differential diagnosis of infectious acute diarrhea in those children demanding hospitalization. Moreover, no advantage of fecal tests over the measurement of CRP was documented.

## Methods

Fifty children with AD were enrolled for the study (26 male, 24 female), including 29 with AD-RV and 21 with AD-SE. The control group consisted of thirty-two healthy subjects (HS; 17 male, 15 female). The setting was a secondary care center. The diagnosis of AD was based on clinical criteria^14^. All patients were hospitalized due to AD at the time of study. Stool samples were collected at admission from all patients whose parents agreed to take part in the study. Collected stool samples were stored at −70°C until analysis. However, fecal M2-PK and calprotectin were exclusively measured in patients with a diagnosis of AD-RV or AD-SE. Rotavirus antigens were detected using an immunochromatographic test (VIKIA Rota-Adeno, Bio-Merieux, Poland). AD-SE was confirmed by stool cultures with a biochemical screen. The diagnostic procedure was carried out according to guidelines approved by the Polish National Institute of Public Health, and was certified by the Polish Centre for Accreditation. The group characteristics are presented in Table 3.

Stool samples were stored at 4° Celsius initially, and at −70° Celsius after they were delivered to the laboratory. The concentration of M2-PK in feces was assessed with the use of a sandwich ELISA kit with monoclonal antibodies (ScheBo Biotech, Giesen, Germany); the values were expressed in U/g. A cut-off value of 4 U/g was employed, in line with manufacturer's recommendations. FC concentrations were measured in μg/mL using the PhiCal ELISA Test (Calpro, Lysaker, Norway); the cut-off value was set at 15 μg/mL. M2-PK and FC levels were measured in the same stool specimens by one technician who did not have information about the diagnosis. CRP was measured with a turbimetric method (Integra 400 plus, Roche Poland).

Statistical analyses were performed using the STATISTICA data analysis software system v. 10 (StatSoft, Inc., Tulsa, United States of America) and Analyse-it v. 2.30 (Analyse-it Software, Leeds, United Kingdom). Comparisons of marker concentrations between the groups were performed using Mann-Whitney U-test. Areas under the receiver operating characteristic curves (ROC), along with sensitivities and specificities, were calculated. For each ROC, the area under the curve (AUC) was described. The AUC's were then compared. The significance level was set at p < 0.05.

The study was conducted in accordance with the Declaration of Helsinki. Parents of all the subjects had expressed their written consent to participation in the study after receiving full information on its scope and purpose. This also applied to subjects who were under age, but at least 16 years old. The study was approved and supervised by the Bioethical Committee at Poznan University of Medical Sciences (decision 1740/04).

## Author Contributions

E.C. envisioned the study, provided material and clinical data, and analyzed the data, J.N. wrote the main manuscript text, J.M. analyzed the data, A.L., A.B., and T.B. helped define the research questions and methodologies, and contributed in data interpretation, J.W. envisioned and supervised the study, and analyzed the data. All authors reviewed the manuscript.

## Figures and Tables

**Table 1:** Pyruvate kinase embryonic isoform M2 (M2-PK) and calprotectin (FC) fecal concentrations in patients with acute diarrhea (AD) caused by infection with rotavirus (AD-RV) and *Salmonella enteritidis* (AD-SE), and in healthy subjects (HS). The data are presented as median [interquartile range]

	M2-PK, U/g	FC, μg/mL
AD-RV	8.7 [2.8–18.8]	20.0 [4.0–40.0]
AD-SE	22.3 [16.3–113.0]	55.0 [22.0–62.5]
HS	*BDL [BDL–1.4]	4.2 [2.3–3.7]

*BDL – below detection limit.

**Table 2:** Sensitivities and specificities of fecal pyruvate kinase embryonic isoform M2 (M2-PK) and calprotectin (FC) in identifying patients with acute diarrhea caused by infection with rotavirus (AD-RV) and *Salmonella enteritidis* (AD-SE) among healthy subjects. Data are presented as sensitivity and specificity (at cut-off value)

	M2-PK		FC	
	standard cut-off	best cut-off	standard cut-off	best cut-off
AD-RV	72.4% and 90.0%	89.7% and 82.9%	55.2% and 97.1%	89.7% and 80.0%
	at 4.0 U/g	at 2.2 U/g	at 15.0 μg/mL	at 3.2 μg/mL
AD-SE	100.0% and 90.0%	95.2% and 100.0%	100% and 97.1%	100% and 97.1%
	at 4.0 U/g	at 10.3 U/g	at 15.0 μg/mL	at 15.0 μg/mL

**Table 3:** Group characteristics. Data for age are presented as median [interquartile range]. AD-RV – rotaviral acute diarrhea, AD-SE – acute diarrhea caused by *Salmonella enteritidis*. HS – healthy subjects

	Number of participants	Age, years	C-reactive protein, mg/L	Leukocytes, G/L
AD-RV	29	2.0 [1.3–3.0]	1.6 [0.9–4.0]	10.0 [8.7–13.4]
AD-SE	21	3.5 [2.6–8.0]	68 [47.2–119]	11.2 [9.7–15.8]
HS	32	2.8 [2.0–4.0]		
